# Psychiatric comorbidity among alcohol-dependent individuals seeking treatment at the Alcohol Rehabilitation Unit, Stikland Hospital

**DOI:** 10.4102/sajpsychiatry.v25i0.1218

**Published:** 2019-04-16

**Authors:** Charnotte M. Gabriels, Muiruri Macharia, Lize Weich

**Affiliations:** 1 Department of Psychiatry, Faculty of Medicine and Health Sciences, Stellenbosch University, Cape Town, South Africa

**Keywords:** Alcohol use disorder, Alcoholism, Alcohol dependence, Alcohol addiction, Comorbidity, Dual diagnosis, Psychiatric illness

## Abstract

**Background:**

International studies have found high rates of psychiatric comorbidity among patients with alcohol use disorders (AUDs) and highlighted the clinical and prognostic implications of this finding. There is a paucity of information with regard to the extent of this problem within the South African context.

**Aim:**

The aim of this study was to investigate the prevalence of psychiatric comorbidity (DSM IV-TR) in treatment-seeking, alcohol-dependent South Africans.

**Setting:**

This study was conducted at the Alcohol Rehabilitation Unit (ARU), Stikland Hospital, Western Cape.

**Methods:**

This cross-sectional study was conducted over a 6-month period. The Mini-International Neuropsychiatric Interview (MINI, version 5) was used to assess psychiatric comorbidity in 101 (male, *n* = 65; 64.5%) alcohol-dependent patients. Interviews were conducted after the first week of admission to ward 13.

**Results:**

Most participants (*n* = 63, 62.4%) had a co-occurring psychiatric disorder, the most common being major depressive (*n* = 30, 29.7%) and anxiety disorders (*n* = 43, 42.6%). Of the anxiety disorders, agoraphobia without a history of panic disorder (*n* = 10, 9.9%) and social phobia (*n* = 10, 9.9%) occurred most frequently, followed by generalised anxiety disorder (*n* = 9, 8.9%) and post-traumatic stress disorder (*n* = 9, 8.9%). Thirteen patients (13%) had a comorbid substance use disorder other than AUD.

**Conclusion:**

The prevalence of psychiatric comorbidity at this unit is high, especially among female patients. The findings emphasise a need to thoroughly assess patients and provide treatment and personnel who can manage the complex needs of a dual diagnosis patient population.

## Introduction

Psychiatric comorbidity, the co-occurrence of multiple mental disorders in an individual, is a well-established phenomenon in people with alcohol use disorders (AUDs).^1,2,3^ Persons with AUD are more likely to have additional psychiatric disorders, the most frequent being mood, anxiety and substance use disorders.^4,5^ Although the precise aetiology is not clear, Koob proposed that the heavy use of alcohol leads to alterations in brain transmitters and hormonal systems which, in turn, might contribute to the development of common psychiatric disorders.^6^ Another explanation for psychiatric comorbidity among patients with AUD is that the psychosocial stressors (legal, financial, interpersonal relationship difficulties) related to alcohol dependence may also predispose the individuals to psychiatric problems.^7^

The presence of additional disorders has implications for the patient, clinician and service provision. Patients with dual diagnoses often have poorer response to treatment, higher rates of dropout, relapse and readmission, and may present with symptoms that are more severe and persistent than other patients.^8,9^ In addition, these patients are generally more impaired and suicidal, have difficulties in maintaining relationships and stable financial profile, have more conflicts with the law and generally make more use of healthcare services.^9,10,11,12^ Therefore, an understanding of psychiatric complications in patients with AUD is important if effective, integrated treatment addressing both comorbidities is to be successfully implemented.

South Africa has a relatively high (30.0%) lifetime prevalence of mental illness, with the Western Cape having the highest rate (39.0%) among the nine provinces.^13,14,15^ Williams et al. reported that the most prevalent categories of lifetime disorders were anxiety (15.8%), substance use (13.3%) and mood disorders (9.8%).^13^ Studies looking at both population and clinical samples in the province have also confirmed the association between psychiatric comorbidity and substance use disorders.^16,17,18,19^

Using results from the South African Stress and Health household survey, Saban et al. found significant associations between substance use and mood and anxiety disorders compared to persons who did not use substances.^16^ Furthermore, when looking at alcohol use, 17.7% of patients with alcohol use met criteria for a lifetime anxiety disorder and 8.2% for a 12-month anxiety disorder, while 11.8% met criteria for a lifetime major depression and 6.1% for a 12-month major depression.^16^ Saban et al. found high rates of antisocial personality and conduct disorders among substance using adolescents and young adults in inpatient treatment,^17^ and Weich et al. found that 51.0% of psychiatric inpatients had a secondary substance use disorder.^18^ Abler et al. investigated alcohol use, post-traumatic stress disorder and depressive symptoms in South African women who attended alcohol-serving venues and described the complex interplay between these three problems.^19^

High rates of psychiatric comorbidity can, therefore, be expected in patients with AUD, yet we could not find any South African reports on the extent of the problem in this patient population. Therefore, the aim of this study was to examine psychiatric comorbidity at the province’s only public alcohol rehabilitation unit (ARU).

## Research methods and design

### Study design

This cross-sectional study was conducted over a 6-month period, between August 2015 and January 2016.

### Study setting

This study was conducted at the ARU, Stikland Hospital, a tertiary psychiatric facility in Western Cape, South Africa.

### Population and sampling

The study sample was drawn from inpatients at the ARU. A total of 159 patients were admitted during the study period. The primary investigator attended the unit weekly and screened all admissions in their second week of treatment for inclusion. Inclusion criteria were that participants had to be at least 18 years of age, clinically stable, willing to give consent and literate enough to complete the questionnaire. We excluded individuals who did not meet eligibility criteria or were unwilling to participate, as well as those who were not present in the unit at the time of screening. In instances where patients were readmitted during the study period, we collected data from only the first admission. A total of 101 patients were recruited.

### Data collection

Data were collected from participants after the first week of admission to limit interference from withdrawal symptoms. A data collection sheet was used to record socio-demographic, admission and general health details. The English version of the Mini-International Neuropsychiatric Interview (MINI, version 5) was used to evaluate previous and current psychiatric disorders.^20^

### Data analysis

Continuous variables were summarised as means and standard deviations, while categorical variables were summarised as count and percentages. Comparisons between variables were made using the *t*-test and Chi-square test for continuous and categorical variables, respectively. Data were analysed using the Statistical Package for Social Sciences version 22 (SPSS 22), and the level of statistical significance was set at *p* < 0.05.

## Ethical consideration

This study was approved by the Health Research Ethics Committee of Stellenbosch University, Cape Town, South Africa (HREC# S14/08/163). All participants included in the analysis gave written informed consent and were treated according to locally and internationally accepted ethical guidelines. Refusal to participate in this study did not impact their treatment.

## Results

### Sample demographics

Of all the participants who were recruited (*N* = 101), 65 (64.4%) were men and the sample had a mean age of 42.5 (standard deviation [s.d.] = 9.9) years (Table 1). The vast majority were single or living alone (84.2%), educated to high school level (grades 8–12; *n* = 73, 72.3%) and unemployed (*n* = 67, 66.3%).

**TABLE 1:** Socio-demographic and clinical characteristics of participants (*N* = 101).

Variable	Overall				Gender									Comorbidity								
					Men				Women				*p*	Yes				No				*p*
	*n*	IQR	%	s.d.	*n*	IQR	%	s.d.	*n*	IQR	%	s.d.		*n*	IQR	%	s.d.	*n*	IQR	%	s.d.	
Comorbidity	63	-	62.4	-	33	-	50.8	-	30	-	83.3	-	0.001*	-	-	-	-	-	-	-	-	-
Mean age	42.5	-	-	9.9	43.4	-	-	9.8	41.0	-	-	10.0	0.245	40.7	-	-	9.3	45.6	-	-	10.3	0.015*
Employed	31	-	30.7	-	26	-	40.0	-	5	-	13.9	-	0.007*	16	-	25.4	-	15	-	39.5	-	0.182
Marital status (single/alone)	85	-	84.2	-	52	-	80.0	-	33	-	91.7	-	0.16	55	-	87.3	-	30	-	78.9	-	0.399
Education (high school, grades 8–12)	73	-	72.3	-	47	-	72.3	-	26	-	72.2	-	0.993	51	-	81.0	-	22	-	57.9	-	0.021*
Mean alcohol debut age	16.0	14.5–19.0	-	-	15.0	14.0–17.0	-	-	15.0	12.8–18.0	-	-	0.119	15.0	13.0–17.8	-	-	15.0	14.0–17.0	-	-	0.940
Mean drug use debut age	17.0	15.0–21.0	-	-	17.0	15.5–20.0	-	-	17.5	15.0–22.8	-	-	0.850	17.5	16.0–21.8	-	-	17.0	15.0–20.0	-	-	0.928
Past rehab (yes)	36	-	35.6	-	25	-	38.5	-	11	-	30.6	-	0.517	26	-	41.3	-	10	-	26.3	-	0.14
Past arrest (yes)	57	-	56.4	-	44	-	67.7	-	13	-	36.1	-	0.003*	33	-	52.4	-	24	-	63.2	-	0.309
FH substance use disorder	67	-	67.0	-	41	-	64.1	-	26	-	72.2	-	0.508	43	-	68.3	-	24	-	64.9	-	0.826
FH psych illness	33	-	32.7	-	17	-	26.2	-	16	-	44.4	-	0.08	23	-	36.5	-	10	-	26.3	-	0.382
Head injury	39	-	38.6	-	23	-	35.4	-	16	-	44.4	-	0.400	27	-	42.9	-	12	-	31.6	-	0.297
Seizure	14	-	13.9	-	8	-	12.3	-	6	-	16.7	-	0.56	11	-	17.5	-	3	-	7.9	-	0.24
Suicidality	41	-	40.6	-	18	-	27.7	-	23	-	63.9	-	0.001*	34	-	54.0	-	7	-	18.4	-	0.001*

FH, family history; s.d., standard deviation; IQR, interquartile range.

* Statistical significance at *p* < 0.05

### Alcohol and drug use

Long-term alcohol use was common in the study population, with 79 (78.2%) patients reporting that they had been drinking excessively for more than 12 months. Most participants (*n* = 47, 46.5%) reported drinking a combination of beer, wine and spirits. Other participants drank beer (*n* = 24, 24.8%) or wine (*n* = 19, 18.8%) as their preferred beverage. Seventy-nine participants (78.2%) smoked cigarettes or used tobacco. The drug most experimented with was cannabis (*n* = 35, 34.65%) followed by a combination of drugs (*n* = 10, 9.90%), stimulants (*n* = 6, 5.94%), mandrax (*n* = 1, 1%), heroin (*n* = 1, 1%) and prescription medication (*n* = 1, 1%).

### Prevalence of comorbid psychiatric disorders

Psychiatric comorbidity was identified in 63 (62.4%) participants, and the proportion was significantly higher in women than in men (83.3% vs. 50.8%, *p* = 0.001). As shown in Table 1, the groups with and without comorbidities did not differ in most of the socio-demographic variables, but the former were significantly more likely to be younger (41 vs. 46 years), educated to high school level (81.0% vs. 57.9%) and admitted to suicidal ideation (54.5% vs. 18.4%). Overall, women were more likely to have suicidal thoughts than men (63.9% vs. 27.7%; *p* = 0.001).

Of those with comorbidity, 28 (44.4%) had one disorder, 35 (55.6%) had 2 or more and none had more than 4 disorders (Table 2).

**TABLE 2:** Number of psychiatric disorders in total population (*N* = 101).

Number of comorbid diagnoses	*n*	%
0	38	37.6
1	28	27.7
2	21	20.8
3	9	8.9
4	5	5.0

### Types of psychiatric comorbidities

Mood and anxiety disorders were the most common comorbidities (Table 3). Major depressive disorder was the predominant mood disorder, while agoraphobia, social phobia and generalised anxiety were the most frequent anxiety disorders. The majority of participants with comorbid psychiatric disorders smoked cigarettes or used tobacco (*n* = 46). Some participants experimented with cannabis (*n* = 23) and stimulants (*n* = 4). Only 13 participants met criteria for other substance use disorders.

**TABLE 3:** Frequencies of different psychiatric disorders among patients with comorbidity (*N* = 63).

Psychiatric diagnosis	Frequency of diagnosis	
	*n*	%
**Mood disorders**	**39**	**61.9**
MDD	30	76.9
Bipolar	8	20.5
Dysthymia	1	2.6
**Anxiety disorders**	**43**	**68.3**
Agoraphobia (no history of PD)	10	23.3
Social phobia	10	23.3
GAD	9	20.9
PTSD	9	20.9
Panic disorders	4	9.4
OCD	1	2.3
**Other substance use disorder**	13	20.6
**Personality disorders**		
Antisocial personality disorder	10	15.9
**Psychotic disorders**	2	3.2
Eating disorders	1	1.6

MDD, major depressive disorder; PD, panic disorder; GAD, generalised anxiety disorder; PTSD, post-traumatic stress disorder; OCD, obsessive-compulsive disorder.

The proportions of men and women with mood and anxiety disorders did not differ significantly (Table 4). Current or past dependence on substances other than alcohol was reported in 13 (12.9%) participants. Furthermore, 10 (9.9%) had antisocial personality disorder and only 3 (3.0%) had a psychotic or eating disorder.

**TABLE 4:** Selected profile of participants with psychiatric comorbidity.

Variable	Overall (*n* = 63)			Gender						*p*
				Men (*n* = 33)			Women (*n* = 30)			
	*n*	%	s.d.	*n*	%	s.d.	*n*	%	s.d.
Mean age in years	40.7	-	9.3	41.4	-	9.6	39.9	-	9.0	0.535
Education (high school)	51	81	-	27	81.8	-	24	80.0	-	0.854
Mean alcohol debut age	17.1	-	5.8	15.5	-	3.5	18.9	-	7.2	0.024
Mean drug debut age	19.5	-	6.3	18.3	-	3.4	20.7	-	8.2	0.265
Suicidality	34	54.0	-	13	39.4	-	21	70	-	0.023
Mood disorder	24	38.1	-	13	39.4	-	11	36.7	-	0.824
Anxiety disorder	30	47.6	-	19	57.6	-	11	36.7	-	0.097
Major depression	37	58.7	-	18	54.5	-	19	63.3	-	0.479
Antisocial	10	15.9	-	7	21.2	-	3	10.0	-	0.224

s.d., standard deviation.

## Discussion

The major findings of this study document the high rates (62.4%) of psychiatric comorbidity at the only public ARU in the Western Cape, South Africa. To our knowledge, this study represents the first analysis of AUD comorbidity with other mental disorders in an alcohol treatment setting in South Africa. Rates reported elsewhere vary widely from 46.0% to 92.0% depending on the settings or patient population, assessment tools used and timing of the diagnostic interviews cognisant of withdrawal effects.^2,4,5,21^ A study conducted in Kerala, India, reported a prevalence of 66.59%, which is comparable to the present study.^22^

Mood and anxiety disorders were the most frequent comorbidities, and more than half of the total sample had two or more individual disorders. The prominence of mood, anxiety, substance use and antisocial disorders in this study is similar to that reported in past studies,^4,5,21,22^ and is consistent with the pattern reported for the general South African population.^13^ Various models have been proposed to explain the high comorbidity between AUDs and mood and anxiety disorders and have included, among others, a causal relationship, cognitive bias or shared risk because of psychopathology during childhood.^23,24,25,26^

Only three cases of psychotic and/or eating disorders were identified in the present study that likely reflects the low community prevalence of these disorders. However, it is possible that patients with AUD with these conditions were receiving treatment elsewhere, that is, within the general psychiatry department or eating disorder unit. Similar to other studies,^27,28^ a substantial proportion of participants (56.0%) in the present study had multiple psychiatric diagnoses, which demonstrates the heterogeneity of comorbidity in AUD populations and emphasises the importance of exhaustive assessment.

The preponderance of males in our sample (64.0%) has also been noted in other treatment-seeking samples.^29^ The observation emphasises the previously acknowledged underuse of alcohol treatment programmes by women, which is often because of factors related to greater stigma associated with alcohol use compared with men as well as socio-economic factors such as child care and concerns about child custody issues.^30,31,32^

However, the proportion of women with comorbidity was much higher than men (83.0% vs. 51.0% in men), with significantly higher rates of mood and anxiety disorders in women. Mood and anxiety disorders are generally more common in women in both alcohol-dependent and nondependent cohorts.^33,34,35,36^ It has been hypothesised that the motivation for drinking in women is more likely to self-treat emotional difficulties, including depression and anxiety, while men are more likely to drink for effect or in response to peer pressure.^34^

A worrisome finding was the high prevalence of suicidality in the entire sample (41.0%) and, particularly, in women with additional psychopathology (70.0%). For alcoholics, being female and having depression are associated with an increased number of suicide attempts.^37^ Out of every 10 women, 8 had an additional psychiatric disorder and 6 of these had suicidal tendencies. Females are, therefore, a particularly vulnerable group in this context and need to be assessed accordingly.

Our participants with comorbidity were younger and likely to have more years of schooling compared to participants without comorbidity. It is possible that patients with comorbidity experience problems from alcohol use sooner and, therefore, present to our service at younger age or are referred sooner. Furthermore, better educated people may be more aware and responsive to their symptoms or may be more resourceful in negotiating access to treatment services and, therefore, more likely to seek treatment.^38^

Overall, psychiatric comorbidity has important clinical implications for patients with AUDs as it generally predicts poorer treatment outcomes.^1,39^ Therefore, the high prevalence found in this study is important because many programmes have limited access to mental health workers. This study highlights the need to employ staff with skills to recognise and treat psychiatric comorbidity in programmes that treat patients with AUD.

This study has several limitations. Firstly, patients who had been admitted but did not participate because they had self-discharged during the first week, who were not present in the unit at the time of recruitment because of a transfer for medical treatment, or who did not meet inclusion criteria may have influenced findings. Secondly, although mood and anxiety disorders (the major comorbid categories in this study) are more likely to precede the onset of alcohol addiction, at least in women,^13^ the precise temporal sequence, aetiology and course of the disorders in the context of the substance use disorder cannot be established in this cross-sectional study. Thirdly, community studies often report lower rates of comorbidity compared to studies involving treatment-seeking patients – a finding interpreted to indicate that the burden of additional comorbidities may be a motivation to seek treatment.^40^ Therefore, the rates of psychiatric disorders reported here, while providing useful indications, may not be representative of community settings. Fourthly, we assessed and reported suicidal risk broadly in a manner that has limited application in clinical practice. Future studies should delineate levels of risk, including suicidal ideation, suicidal behaviour and impulsivity, to provide more useful information for therapy and risk stratification.

## Conclusion

The results indicate that psychiatric multi-comorbidity is common at this ARU, particularly among the women. This study underlines the importance of screening for suicidality and psychiatric disorders, and the need for subsequent appropriate management as an essential part of treatment planning to enhance treatment outcomes in patients with AUDs. Targeted interventions aimed at preventing and treating these disorders in women should also be considered a priority.
